# A Fast Terahertz Imaging Method Using Sparse Rotating Array

**DOI:** 10.3390/s17102209

**Published:** 2017-09-26

**Authors:** Yanwen Jiang, Bin Deng, Yuliang Qin, Hongqiang Wang, Kang Liu

**Affiliations:** College of Electronic Science and Engineering, National University of Defense Technology, Changsha 410073, China; dengbin@nudt.edu.cn (B.D.); qinyuliang@nudt.edu.cn (Y.Q.); oliverwhq@tom.com (H.W.); liukang1117@nudt.edu.cn (K.L.)

**Keywords:** sparse array optimization, spectral support, synthetic aperture radar, terahertz imaging

## Abstract

For fast and standoff personal screening, a novel terahertz imaging scheme using a sparse rotating array is developed in this paper. A linearly sparse array is designed to move along a circular path with respect to an axis perpendicular to the imaging scenario. For this new scheme, a modified imaging algorithm is proposed based on the frequency-domain reconstruction method in circular synthetic aperture radar. To achieve better imaging performance, an optimization method of the sparse array is also proposed, according to the distribution of the spectral support. Theoretical and numerical analysis of the point spread function (PSF) is provided to demonstrate the high-resolution imaging ability of the proposed scheme. Comprehensive simulations are carried out to validate the feasibility and effectiveness of the array optimization method. Finally, the imaging results of a human-scattering model are also obtained to further demonstrate the good performance of this new imaging scheme and the effectiveness of the array optimization approach. This work can facilitate the design and practice of terahertz imaging systems for security inspection.

## 1. Introduction

Due to the increasing threat of terrorism, security inspection has been becoming increasingly important at many high-security facilities, including airports and railway stations. Generally, effective detection instruments, like X-ray imagers, metal detecting gates and hand-held metal detectors, are widely applied for security inspection. However, for personal screening applications, the X-ray imager cannot be an acceptable choice because of its harmful effects on body health. Metal detectors are not suitable due to their dependence on human assistance, which leads to low efficiency. With their safety characteristics and short data acquisition time, terahertz (THz) and millimeter wave (MMW) technologies have been widely researched for security applications [1,2,3,4]. Moreover, THz waves and MMWs have penetration capability, and can achieve high spatial imaging resolution, which enables them to display good performance in the detection of concealed dangerous objects. Therefore, THz and MMW imaging is an alternative and effective means for personal screening.

Hitherto, a number of THz and MMW imagers have been developed for personal screening, which can be divided into two types: mechanical scanning [5,6,7,8] and multistatic arrays [9,10,11]. The mechanical scanning architecture is usually implemented by a linearly moving process or fast rotating reflector. In particular, the linearly moving process requires either a single antenna scan, which lasts at least several minutes and results in low efficiency, or a linear array scan, which is very expensive [5]. The optical reflector used for fast rotating usually requires a high-precision manufacturing technique, and is also very expensive [6,7]. In addition, despite the very fast imaging speed, multistatic architectures based on thousands of antennas are bulky, complicated, and costly for practical implementation [9,11]. Recently, an imaging method has been proposed based on the mechanical scanning technique and multistatic arrays [12], which significantly reduces the time cost compared to the traditional mechanical scanning method, but still requires hundreds of antennas. Hence, to overcome the tradeoff between system cost and imaging speed, THz and MMW imagers still need further investigation for wide application under practical conditions.

In this paper, a new terahertz imaging method for standoff personal screening is proposed based on the circular synthetic aperture radar (SAR) configuration and the sparse array technique. The circular scanning method ensures nonstop scanning with fast speed and high imaging resolution, and the sparse array technique, which only needs several antennas, reduces the system cost. The rest of this paper is organized as follows. In Section 2, the new terahertz imaging scheme is described in detail, and the spectral support and the point spread function (PSF) are analyzed. A modified imaging algorithm is developed based on the circular SAR imaging technique [13,14]. In Section 3, an optimization method for the sparse array based on the distribution of the spectral support is proposed, in order to achieve better imaging performance. Different from the existing array optimization method [15,16], the proposed array optimization method is based on the distribution of the spectral support and the imaging geometry. Simulations are performed to validate the effectiveness of this proposed method. In Section 4, simulations of the imaging of a human-scattering model are carried out to further demonstrate the good performance of the new imaging scheme. Results verify the effectiveness of the proposed imaging algorithm and the array optimization method. The conclusions are drawn in Section 5.

## 2. The Proposed Imaging Scheme and Algorithm

### 2.1. Description of the Imaging Model

**Figure 1:**
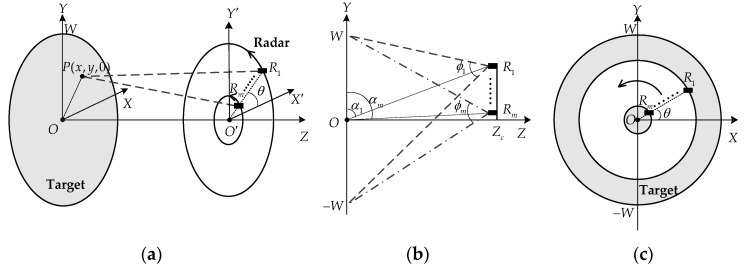
Observation geometry of the proposed imaging method: (**a**) Perspective view; (**b**) Front view; (**c**) Right side view.

The observation geometry of the proposed imaging scheme is shown in Figure 1, where the front view displayed in Figure 1b is rotationally symmetric about the *OZ* axis. The target coordinate system *OXYZ* is fixed. The radar, consisting of *m* self-transceiver antennas, moves along a circular path on the vertical plane $z=Z_{c}$, i.e., the $X^{\prime }O^{\prime }Y^{\prime }$ plane, where $Z_{c}$ is the range between the radar and the target in the horizontal direction. Thus, the radius of the circular path for each antenna is $R_{i}\in [R_{m},R_{1}],\;i=1,\;\ldots ,\;m$, and the coordinate of the ith antenna in the spatial domain can be denoted by $\left(R_{i}\cos\theta ,\;R_{i}\sin\theta ,\;Z_{c}\right)$, where θ∈[0,2π) is the azimuthal angle. It can be seen from Figure 1a that the circular synthetic aperture achieved by one antenna is similar to the synthetic aperture of the circular SAR [14,17]. In Figure 1b, $\alpha_{i}$ represents the side angle of the ith antenna with respect to the origin O, which is equal to $\arctan\;(Z_{c}/R_{i})$. It should be noted that all the antennas rotate around the same center $\left(0,0,Z_{c}\right)$ and the same azimuthal angle θ, but with different radii $R_{i}$. As the radar moves along the circular path, the beams of all the antennas are spotlighted on a disk with radius W centered at the origin O on the XOY plane, where W indicates the radius of the imaging scenario. It can be seen from Figure 1b that the half-power beamwidth $\phi_{i}$ of the ith antenna can be denoted by $\phi_{i}=\arctan\;[(W-R_{i})/Z_{c}]+\arctan\;[(W+R_{i})/Z_{c}]$ [13]. Accordingly, the half-power beamwidth ϕ of all the antennas should satisfy the condition

(1) $$\phi \geq \max\left\{\phi_{1},\;\ldots ,\;\phi_{m}\right\}=\max\left\{\arctan\;\left(\frac{W-R_{i}}{Z_{c}}\right)+\arctan\;\left(\frac{W+R_{i}}{Z_{c}}\right),\;i=1,\;\ldots ,\;m\right\}$$

It is assumed that the antennas transmit the linear frequency modulation (LFM) signal in time sequence. When the LFM signal is transmitted by the *i*th antenna, the *i*th antenna itself is used to receive the echo signal, which can be defined by

(2) $$s_{i}(k,\theta )=\int \int g(x,y)\times e^{-j2k\sqrt{{\left(x-R_{i}\cos\theta \right)}^{2}+{\left(y-R_{i}\sin\theta \right)}^{2}+Z_{c}^{2}}}\;dxdy$$

where g(x,y) denotes the reflectivity function and k=2πf/c is the wavenumber. $f\in [f_{c}-B/2,\;f_{c}+B/2]$, where $f_{c}$ is the center frequency. B is the bandwidth and c is the light speed in the free space.

### 2.2. Analysis of the Spectral Support and PSF

According to Equation (2) the phase trace of the ith antenna response is $\Omega_{i}=-2k\sqrt{{\left(x-R_{i}\cos\theta \right)}^{2}+{\left(y-R_{i}\sin\theta \right)}^{2}+Z_{c}^{2}}$. The spatial frequency along the *X* and *Y* direction on the XOY plane can then be defined by the derivative of $\Omega_{i}$, i.e.,

(3) $$\begin{array}{c} k_{x}=\frac{\partial \Omega_{i}}{\partial x}=-2k\frac{x-R_{i}\cos\theta }{\sqrt{{\left(x-R_{i}\cos\theta \right)}^{2}+{\left(y-R_{i}\sin\theta \right)}^{2}+Z_{c}^{2}}}\\ k_{y}=\frac{\partial \Omega_{i}}{\partial y}=-2k\frac{y-R_{i}\sin\theta }{\sqrt{{\left(x-R_{i}\cos\theta \right)}^{2}+{\left(y-R_{i}\sin\theta \right)}^{2}+Z_{c}^{2}}} \end{array}$$

It can be known from Equation (3) that the spectral support of the imaging scheme is spatially variant, because the values of $k_{x}$ and $k_{y}$ are dependent on the location of the target P(x,y,0). In particular, for the target located at origin (0,0,0), the corresponding spatial frequency can be written as

(4) $$\begin{array}{c} k_{x}=2k\cos\alpha_{i}\cos\theta \\ k_{y}=2k\cos\alpha_{i}\sin\theta \end{array}$$

where $\cos\alpha_{i}=R_{i}/\sqrt{Z_{c}^{2}+R_{i}^{2}}$.

**Figure 2:**
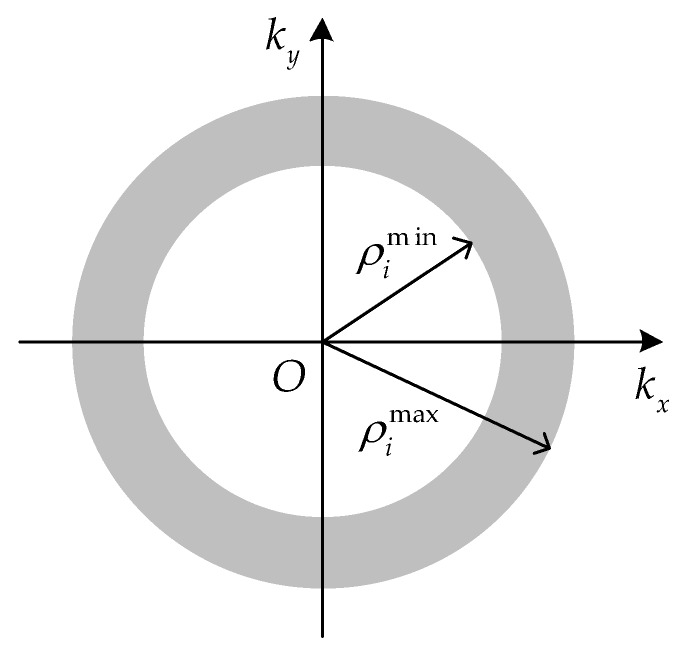
The spectral support of imaging with one antenna.

Denote the spatial wavenumber as $\rho_{i}=\sqrt{k_{x}^{2}+k_{y}^{2}}=2k\cos\alpha_{i}$. When a signal with wide bandwidth is used and the condition θ∈[0,2π) is satisfied, the two-dimensional spectral support of the *i*th antenna imaging is an annulus in Figure 2, and the radii are $\rho_{i}^{\min}=2k_{\min}\cos\alpha_{i}$ and $\rho_{i}^{\max}=2k_{\max}\cos\alpha_{i}$, respectively. To obtain a better visual effect, only one annulus is displayed as an example in Figure 2, which represents the spectral support of one antenna. It can be noted that the spectral support of any other antenna is a similar annulus with a different radius.

Accordingly, the PSF of the target located at the center point (0,0,0) in the spatial domain is [13,18]:

(5) $${\mathrm{psf}}_{i}(x,y)=\frac{\rho_{i}^{\max}J_{1}(\rho_{i}^{\max}r)-\rho_{i}^{\min}J_{1}(\rho_{i}^{\min}r)}{r}$$

where $r=\sqrt{x^{2}+y^{2}}$, $J_{1}$ is the first order Bessel function of the first kind. The radial resolution becomes $\pi /\rho_{i}^{\max}$ under a limit condition $\rho_{i}^{\min}=0$. Hence, the radial resolution is approximately $a_{0}\pi /\rho_{i}^{\max}$, where $1\leq a_{0}\leq 2$ [14]. The spectral support of the proposed imaging scheme with m antennas is a combination of m annuluses, shown in Figure 2, and thus the final PSF can be written as the accumulation of Equation (5).

(6) $$\mathrm{PSF}(x,y)=\sum_{i=1}^{m}{\mathrm{psf}}_{i}(x,y)=\sum_{i=1}^{m}\frac{\rho_{i}^{\max}J_{1}(\rho_{i}^{\max}r)-\rho_{i}^{\min}J_{1}(\rho_{i}^{\min}r)}{r}$$

As $\cos\alpha_{i}$ is proportional to $R_{i}$, the best radial resolution depends on $a_{0}\pi /\rho_{1}^{\max}$. The behavior of the point spread function and its spatial resolution depends on the bandwidth of the radar signal and the array configuration. Hence, the array optimization needs to be studied to further improve the imaging performance.

### 2.3. Modified Imaging Algorithm

According to the array configuration, in order to meet the requirements of real-time imaging for personal screening, a modified imaging algorithm is proposed based on the frequency-domain reconstruction method described in [13,14]. The circular SAR reconstruction method is based on Fourier analysis and the slant plane Green’s function, which is free of approximation. Specifically, the slant plane circular SAR phase history is firstly transmitted into the ground plane phase history, and then the target area reconstruction based on the ground plane circular SAR data is conducted. Consequently, as the observation geometry of the new THz imaging scheme is vertical to that of circular SAR, the proposed imaging algorithm in this paper mainly includes two steps, i.e., the slant plane to vertical plane transformation and the vertical plane reconstruction.

In general, the interpolation process is essential in the target reconstruction of the circular SAR, which transforms the target spectrum from polar coordinate form to rectilinear coordinate form. However, the calculation volume and time consumption for the interpolation algorithm is enormous, and this would be multiplied with the array configuration in the proposed imaging scheme. Hence, to meet the real-time requirement of personal screening, the Non-Uniform Fast Fourier Transform (NUFFT) is used in the proposed imaging algorithm. The NUFFT algorithm possesses a particularly fast and simple implementation [19,20], which can substitute the interpolation processing and the two-dimensional inverse Fourier transform. The imaging procedure is described in detail as follows.

Firstly, the slant plane to vertical plane transformation is performed on the echo of each antenna separately. In particular, the processing of the *i*th antenna can be achieved by

(7) $$s_{i}^{v}(\rho_{i},\theta )=\int_{k}\Lambda^{*}(\rho_{i},k)s_{i}(k,\theta )dk$$

where

(8) $$\Lambda (\rho_{i},k)=W_{f}(\rho_{i},k)\times e^{-j\sqrt{4k^{2}-{\left(\rho_{i}\right)}^{2}}Z_{c}}$$

Different from the target reconstruction approach in circular SAR, the proposed imaging method concentrates on a fixed scenario with constant radius. The window function $W_{f}(\rho_{i},k)$ in the polar spatial frequency domain is defined as

(9) $$W_{f}(\rho_{i},k)=\{\begin{array}{c} 1\;2k\cos\alpha_{i}^{\max}\leq \rho_{i}\leq 2k\cos\alpha_{i}^{\min}\\ 0\;\mathrm{otherwise} \end{array}$$

where $\alpha_{i}^{\max}=\arctan\;[Z_{c}/(R_{i}-W)]$ and $\alpha_{i}^{\min}=\arctan\;[Z_{c}/(R_{i}+W)]$ are the maximum and minimum side angles of the *i*th antenna with respect to each edge of the scenario, respectively.

The second step, i.e., the vertical plane reconstruction, is performed by

(10) $$F_{i}(\rho_{i},\xi )=S_{i}^{v}(\rho_{i},\xi )\Gamma_{i}^{*}(\rho_{i},\xi )$$

where ξ is the Fourier counterpart domain of θ (Fourier series domain) and ${ℱ}_{\left(\theta \right)}[\;]$ denotes one-dimensional Fourier transform with respect to θ. $S_{i}^{v}(\rho_{i},\xi )={ℱ}_{\left(\theta \right)}[s_{i}^{v}(\rho_{i},\theta )]$. $\Gamma_{i}^{*}(\rho_{i},\xi )$ is the conjugate of $\Gamma_{i}(\rho_{i},\xi )$, $\Gamma_{i}(\rho_{i},\xi )={ℱ}_{\left(\theta \right)}[e^{-j\rho_{i}R_{i}\cos\theta }]$.

**Figure 3:**
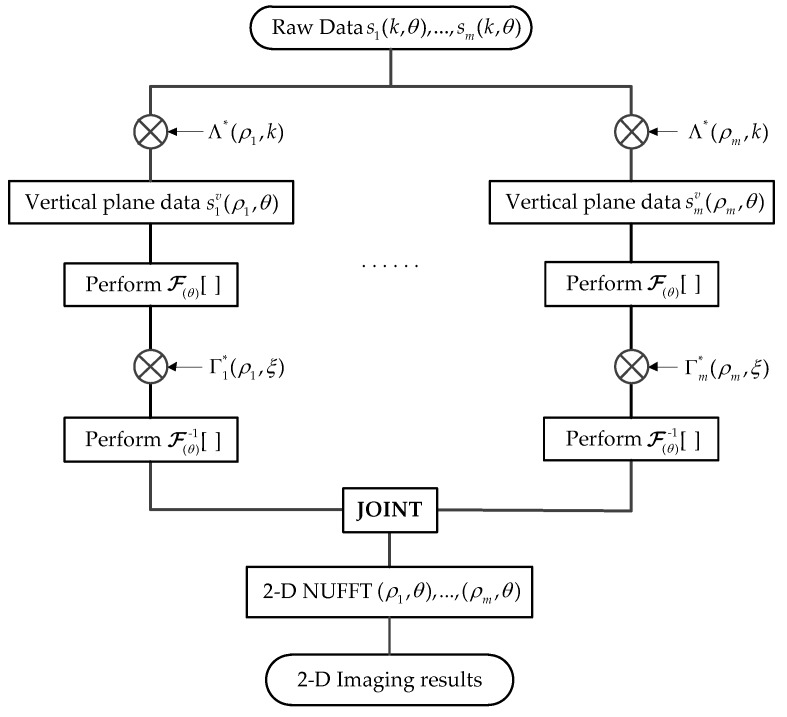
Flowchart of the proposed imaging algorithm.

Therefore, the target function in the polar spatial frequency domain can be obtained from the inverse transformation $F_{i}(\rho_{i},\theta )={ℱ}_{\left(\theta \right)}^{-1}[F_{i}(\rho_{i},\xi )]$. Then, the imaging result with high resolution can be achieved through the joint NUFFT processing of $F_{1}(\rho_{1},\theta ),\;\ldots ,\;F_{m}(\rho_{m},\theta )$. Moreover, the flowchart of the modified imaging algorithm for the proposed imaging method is illustrated in Figure 3.

## 3. Optimization Method of Sparse Array

### 3.1. Array Optimization Method

**Figure 4:**
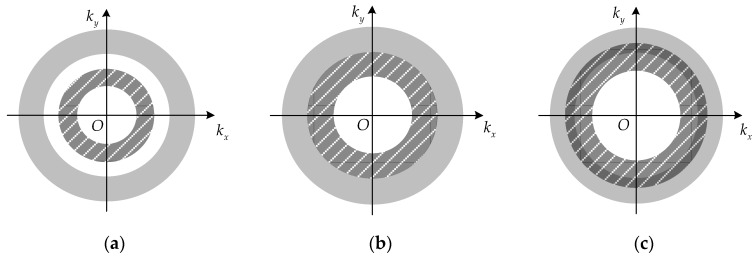
The distribution of two antennas’ spectral supports: (**a**) Separate; (**b**) Adjacent; (**c**) Overlapping.

According to the analysis of PSF in Section 2.2, the spatial resolution of the proposed new imaging method is dependent on the array configuration. Hence, the optimization method of the sparse array is studied here to achieve better imaging performance. The spectral support of the proposed imaging scheme is the combination of m annuluses shown in Figure 2, and the radii are decided by the parameters $R_{1},\;\ldots ,\;R_{m}$. Generally, the spatial distribution of the two antennas’ spectral supports have three types of relationships, i.e., separate, adjacent and overlapping, shown in Figure 4 (each color region indicates the spectral support of one antenna). The values of $R_{i}$ of the outside annuluses remain invariant and have the same value. However, the corresponding $R_{i+1}$ of the inside annulus increases gradually from Figure 4a–c.

Based on geometric knowledge, the area of annulus is proportional to $\cos^{2}\alpha_{i}$ ($\cos^{2}\alpha_{i}\propto R_{i}$), which indicates that the area of the spectral support increases with an increase of $R_{i}$. Hence, the second type of the spatial distribution in Figure 4b has the largest area.

According to the properties of Fourier transform, the imaging resolution increases with the increase of the width of the spectral support [21]. With the same value of $R_{i}$, the imaging resolutions corresponding to Figure 4a–c are the same in theory. However, the density and gap of the spectral support are associated with the side-lobe level, and a lower side-lobe can be obtained when the gap is smaller. Therefore, the array configuration corresponding to the second type of spatial distribution can be used to achieve better imaging performance.

In Figure 4, the radii of the outside annuluses are $2k_{\min}\cos\alpha_{i}$ and $2k_{\max}\cos\alpha_{i}$, and the radii of the inside annuluses are $2k_{\min}\cos\alpha_{i+1}$ and $2k_{\max}\cos\alpha_{i+1}$. Therefore, the spatial distribution of two antennas’ spectral support in Figure 4b can be expressed by:

(11) $$2k_{\min}\cos\alpha_{i}=2k_{\max}\cos\alpha_{i+1}$$

Substitute $\alpha_{i}$ with $\arctan\;(Z_{c}/R_{i})$ and replace $\alpha_{i+1}$ with $\arctan\;(Z_{c}/R_{i+1})$. Then, the relationship between $R_{i+1}$ and $R_{i}$ can be given by:

(12) $$f_{\max}\frac{R_{i+1}}{\sqrt{R_{i+1}^{2}+Z_{c}^{2}}}=f_{\min}\frac{R_{i}}{\sqrt{R_{i}^{2}+Z_{c}^{2}}}$$

The value of $R_{i+1}$ is decided by the parameters $R_{i}$, f and $Z_{c}$, which indicate that the optimization of the sparse array should be conducted with the constraints of system parameters. When the system parameters are fixed, the value of $R_{i+1}$ should be accurately obtained according to Equation (12), which will lead to better imaging performance.

### 3.2. Simulation Results for Array Design

In this section, simulations are performed to show the advantage and effectiveness of the proposed array optimization method. According to the current device level of the terahertz radar, the frequency of the transmitted LFM signal is from 210 GHz to 230 GHz. The radius of the imaging scenario is set as 1 m, which is fit for a human being’s height. The radar system works at a standoff range 3 m from the imaging scene. According to the theoretical analysis above, the best spatial resolution $a_{0}\pi /\rho_{1}^{\max}$ is determined by the maximum value of the antenna rotation radius. Thus, $R_{1}$ is set as 0.6 m to achieve the theoretical imaging resolution 0.0017 m~0.0034 m. The main simulation parameters are listed in Table 1.

To simplify the analysis, two antennas in radial direction are used in the simulations, and the setup of rotation radii is displayed in Table 2. The values of $R_{2}$ are set differently for comparison, resulting in a distribution of the spectral support corresponding to the three types of relationship in Figure 4. The radius $R_{2}$ = 0.546 m of Type IV is calculated according to Equation (12). Additionally, a simulation using a single antenna with radius 0.6 m is also performed.

**Figure 5:**
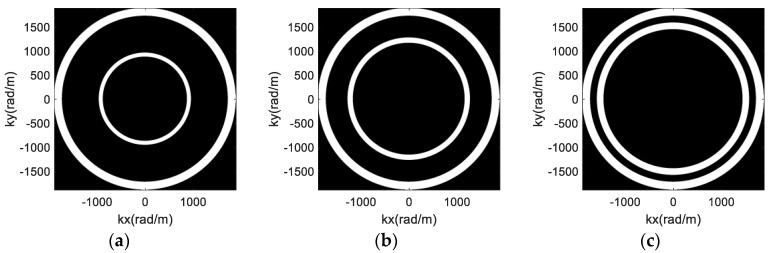

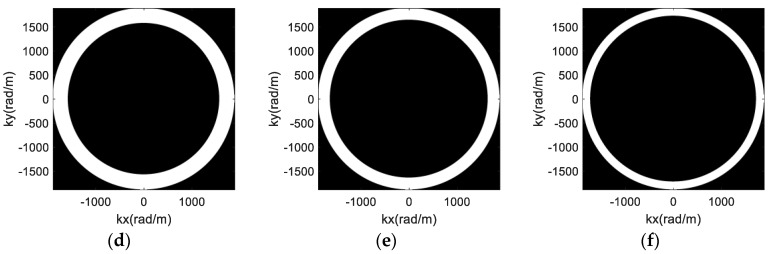
The spectral support of two antennas: (**a**) Type I; (**b**) Type II; (**c**) Type III; (**d**) Type IV; (**e**) Type V; (**f**) Type VI.

**Figure 6:**
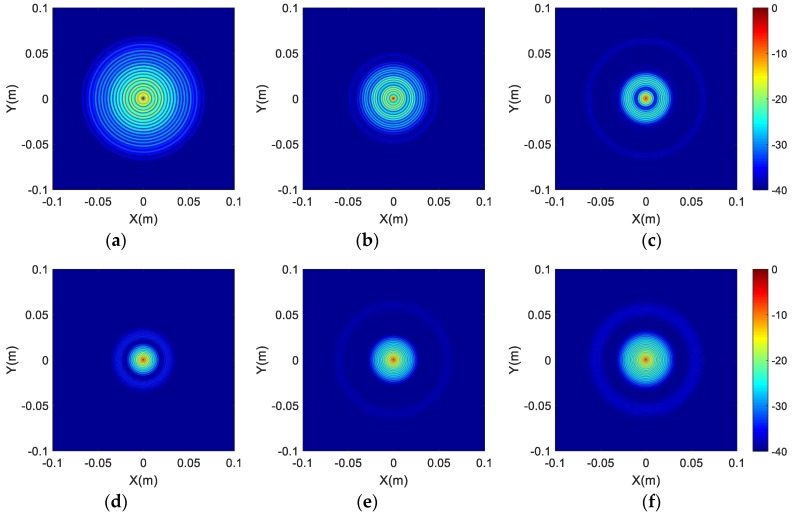
The two-dimensional imaging results at (0, 0): (**a**) Type I; (**b**) Type II; (**c**) Type III; (**d**) Type IV; (**e**) Type V; (**f**) Type VI.

**Figure 7:**
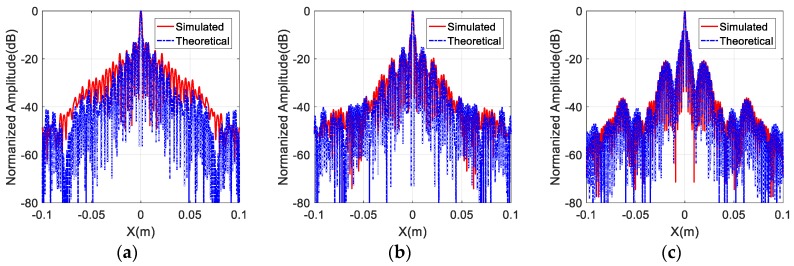

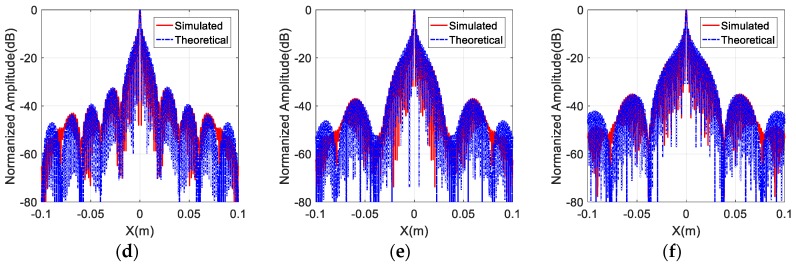
Comparison of the theoretical and simulated PSF: (**a**) Type I; (**b**) Type II; (**c**) Type III; (**d**) Type IV; (**e**) Type V; (**f**) Type VI.

The spectral supports of the different array configurations are displayed in Figure 5. To compare the imaging performance of different array configurations sufficiently, the two-dimensional imaging results at (0,0) and the one-dimensional imaging results are also shown in Figure 6 and Figure 7, respectively. Furthermore, quantitative analysis of the imaging performance is performed, and is listed in Table 3. The meaning and definition of each parameter in Table 3 is explained below.

The area percentage is defined as the ratio of the area of array’s spectral support to the area defined by $k_{x}\in [-2k\cos\alpha_{1},\;-2k\cos\alpha_{1}]$, $k_{y}\in [-2k\cos\alpha_{1},\;-2k\cos\alpha_{1}]$, i.e., the ratio of the area in white color to the area in black color in Figure 5. Obviously, the area percentage is associated with the image side-lobe level. The two-dimensional integral side-lobe ratio (ISLR) is defined as the ratio of the power of the side-lobe to that of the main lobe, which reflects the focusing performance of the image and the lower value represents the better performance. Image entropy has been successfully applied to evaluate the quality of SAR or inverse SAR (ISAR) images [22,23]. A canonical definition of the image entropy is [24]

(13) $$\mathrm{En}=\iint -H(x,y)\times \ln H(x,y)dxdy,\;H(x,y)=\frac{{\left|h(x,y)\right|}^{2}}{\iint {\left|h(x,y)\right|}^{2}dxdy}$$

where H(x,y) is the normalized image power density, h(x,y) depicts the reconstructed reflectivity function of target. However, the imaging results obtained in this paper are discretized images composed of discrete grids; thus, the discretized expression of Equation (13) can be written as

(14) $$\mathrm{En}=-\sum_{p=1}^{P}\sum_{q=1}^{Q}H(p,q)\times \ln H(p,q),\;H(p,q)=\frac{{\left|h(p,q)\right|}^{2}}{\sum_{p=1}^{P}\sum_{q=1}^{Q}{\left|h(p,q)\right|}^{2}}$$

where p and q are the discretized pixels of the imaging result, and P and Q represent the total number of pixels in each row and each column. The two-dimensional image entropy represents the quality of the imaging result, and a smaller value corresponds to a better imaging performance. The values of ISLR and image entropy can be calculated based on Figure 6. Moreover, the 3 dB resolution of the imaging results can be represented by the impulse response width (IRW), which can be obtained from Figure 7.

It can be seen from Figure 5a–c that the spectral support of the previous third array configurations have a separated distribution relationship, and the width of the gap in each annulus decreases from Figure 5a–c. In Table 3, the IRWs of Type I, Type II and Type III are larger than those of the last three array configurations, which indicates that the gap in the spatial frequency domain may degrade the imaging resolution. According to the quantitative analysis of the array configurations of Type I, Type II and Type III, the values of ISLR, image entropy and IRW decrease, which demonstrates that the quality of the imaging results gradually improves from Type I to Type III. Hence, it can be concluded that the narrower the gap in the spatial frequency domain, the better the imaging performance that can be achieved.

Figure 5d represents the adjacent distribution of the spectral support, which corresponds to the optimized array configuration (Type IV). In Figure 5e, the overlapped distribution of spectral support is displayed with respect to Type V. The spectral support of imaging with one antenna located at *R*_1_ = 0.6 m is depicted in Figure 5f. Comparing the imaging results of the six types of array configurations, the ISLR of Type VI has the largest value, which indicates that the obtained image using single antenna has poor imaging performance with higher side-lobe level and lower image-focusing ability. The image entropy of Type I and Type II is larger than that of Type VI, which is caused by the lower IRWs of Type I and Type II. The more antennas that are used, the better the imaging performance that can be achieved. Hence, the advantages of the array configuration for the proposed imaging scheme can be recognized.

According to the results of the area percentage listed in Table 3 and the spectral support shown in Figure 5, the spectral support of the optimized array (Type IV) has the maximum area among the six array configurations. Moreover, the ISLR, image entropy and IRW of the optimized array’s imaging result have the minimum values in all imaging results, which indicates that the optimized array configuration can be used to achieve the best imaging performance with lower and fewer side-lobes. In addition, the best imaging performance of the optimized array configuration can also be recognized with the best visual effect in Figure 6. Therefore, we can draw a conclusion that the proposed optimization method is feasible and effective.

In addition, the theoretical PSF according to Equation (6) is also calculated, and is shown in Figure 7. It can be seen from Figure 7 that the main-lobes of the simulated PSF and the theoretical PSF almost have the same width, despite the different distribution of the antennas. Additionally, the side-lobes of the simulated PSF and the theoretical PSF are more similar when the spectral support is more intense. Thus, the effectiveness of the proposed imaging algorithm is validated.

## 4. Imaging Results and Analysis

**Figure 8:**
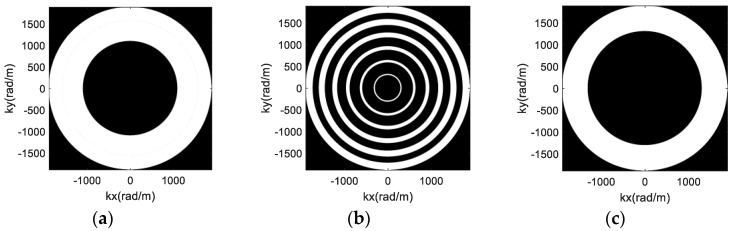
The spectral supports of different array configuration: (**a**) Optimized array; (**b**) Uniform array 1; (**c**) Uniform array 2.

Based on the proposed array optimization method, the imaging results of the human-scattering model are provided to show the advantages of the proposed imaging scheme for personal screening. According to Equation (12), the rotation radii of six antennas are 0.377 m, 0.413 m, 0.453 m, 0.497 m, 0.546 m, and 0.6 m. Two uniform array configurations are considered for comparison, denoted by uniform array 1 and uniform array 2. The rotation radii of uniform array 1 are 0.1 m, 0.2 m, 0.3 m, 0.4 m, 0.5 m and 0.6 m, and the rotation radii of uniform array 2 are 0.45 m 0.48 m, 0.51 m, 0.54 m, 0.57 m and 0.6 m. The spectral supports of the three array configurations are shown in Figure 8. It can be seen from Figure 8b,c that the spectral supports of the two uniform array configurations possess separated and overlapped distribution relationships, respectively. The area percentages of Figure 8 are 52.03%, 33.39% and 41.07%, which indicate that the optimized array has the largest area of spectral support.

**Figure 9:**
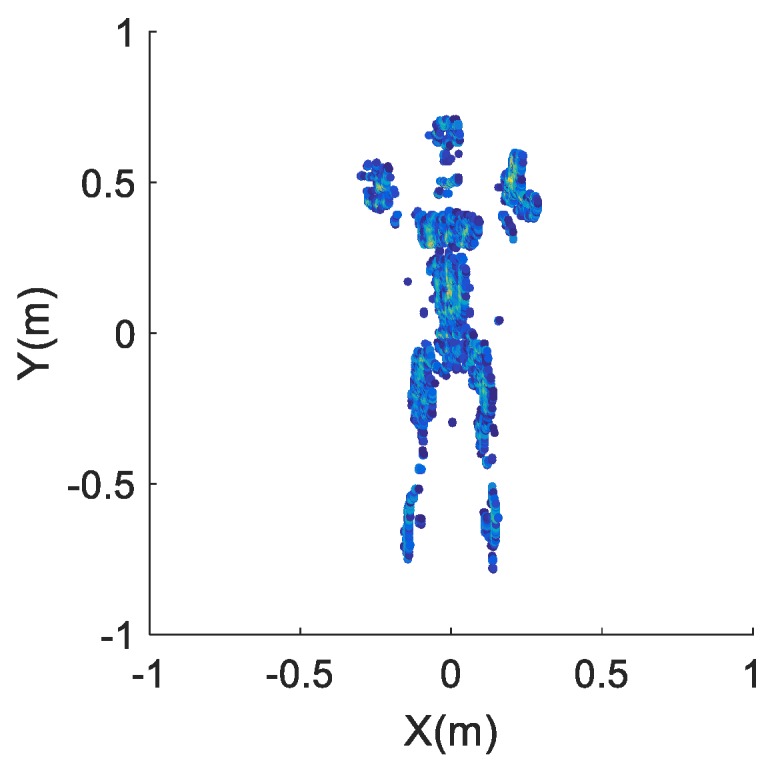
Scattering model of human.

**Figure 10:**
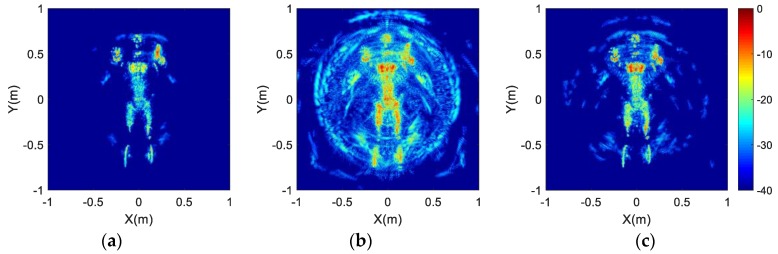
Imaging results of the human-scattering model: (**a**) Optimized array; (**b**) Uniform array 1; (**c**) Uniform array 2.

The reflection distribution of the human-scattering model used is displayed in Figure 9, which contains 3453 scatters with 0.005 m spacing. The imaging results of the three arrays are achieved in Figure 10. The image entropy of the three sub-images in Figure 10 are 11.7326, 12.9384 and 11.9848, which indicate that the array optimization procedure leads to better image quality. Moreover, it can be seen from the imaging result in Figure 10a that the profiles and details of the human model are quite clear. Thus, the proposed new imaging scheme can be used for security inspection.

In this paper, a desktop computer with Intel(R) Core(TM) i5-4460U CPU @ 3.2 GHz and 8 GB RAM is used for the imaging simulation. Through the processing of the 3D data $N_{f}\times N_{\theta }\times m$ (2001 × 3600 × 6), imaging results of 2 × 2 m^2^ with 0.001 m spacing can be achieved in 30 s. Accordingly, the time consumption of the imaging processing can be reduced to several seconds with advanced computer technology, which meets the requirements of the real-time imaging. Hence, the proposed new imaging scheme is suitable for standoff personal screening.

## 5. Conclusions

To conclude, a fast terahertz imaging method based on a sparse rotating array has been proposed in this paper. This new imaging scheme can be used to achieve high imaging resolution, fast imaging speed and low system cost, which is an effective and acceptable terahertz imager for standoff personal screening. A modified imaging algorithm based on the circular SAR reconstruction method was developed for this new imaging scheme, which was validated by analyzing the PSF. Moreover, an optimization method of the sparse array was proposed. Based on the proposed optimization method, a sparse array was obtained and the imaging results of a human-scattering model were achieved, which validated the good performance and merits of the proposed imaging scheme.

## Figures and Tables

**Table 1:** Simulation parameters.

Parameters	Numerical Value
Center frequency $f_{c}$	220 GHz
Bandwidth B	20 GHz
Maximum radius of antenna $R_{1}$	0.6 m
Horizontal range $Z_{c}$	3 m
Imaging scene radius W	1 m
Sampling interval of f	0.01 GHz
Sampling interval of θ	0.1°
Sampling numbers of frequency $N_{f}$	2001
Sampling numbers of azimuthal angle $N_{\theta }$	3600

**Table 2:** Different array configurations.

No.	Type I	Type II	Type III	Type IV	Type V	Type VI
$R_{1}$ (m)	0.6	0.6	0.6	0.6	0.6	0.6
$R_{2}$ (m)	0.3	0.4	0.5	0.546	0.57	

**Table 3:** Quantitative comparison of the different distribution antennas.

No.	Type I	Type II	Type III	Type IV	Type V	Type VI
Area percentage	16.43%	18.99%	22.24%	23.95%	19.22%	13.06%
ISLR (dB)	9.0674	7.5736	7.3180	6.7343	8.4933	9.9953
Image entropy	12.8088	12.0532	11.3908	10.5492	11.1750	11.7455
IRW (m)	0.0019	0.0017	0.0015	0.0013	0.0013	0.0013
